# Nitric oxide induced by Indian ginseng root extract inhibits Infectious Bursal Disease virus in chicken embryo fibroblasts in vitro

**DOI:** 10.1186/s40781-017-0156-2

**Published:** 2018-01-08

**Authors:** Bhaskar Ganguly, Vijaypillai Umapathi, Sunil Kumar Rastogi

**Affiliations:** 1 Animal Biotechnology Center, Department of Veterinary Physiology and Biochemistry, College of Veterinary & Animal Sciences, G. B. Pant University of Agriculture & Technology, Pantnagar, 263145 India; 2 Clinical Research Division, Ayurvet Limited, Katha, 173205 India; 3 Division of Animal Biochemistry, FMD Research Laboratory, Indian Veterinary Research Institute, Hebbal, Bengaluru, 560024 India

**Keywords:** Aminoguanidine, Infectious Bursal Disease virus, Nitric oxide, Pathogenesis, *Withania somnifera*

## Abstract

Infectious Bursal Disease is a severe viral disease of chicken responsible for serious economic losses to poultry farmers. The causative agent, Infectious Bursal Disease virus, is inhibited by nitric oxide. Root extract of the Indian ginseng, *Withania somnifera*, inhibits Infectious Bursal Disease virus in vitro. Also, *Withania somnifera* root extract is known to induce nitric oxide production in vitro. Therefore, the present study was undertaken to determine if the inhibitory activity of *Withania somnifera* against Infectious Bursal Disease virus was based on the production of nitric oxide. We show that besides other mechanisms, the inhibition of Infectious Bursal Disease virus by *Withania somnifera* involves the production of nitric oxide. Our results also highlight the paradoxical role of nitric oxide in the pathogenesis of Infectious Bursal Disease.

## Introduction

Infectious Bursal Disease (IBD) is an acute, highly contagious, and immunosuppressive disease of young chicken, caused by double-stranded RNA virus belonging to the genus *Avibirnavirus* of family *Birnaviridae*. It is characterized by the destruction of dividing lymphoid cells in the bursa of Fabricius causing cytolysis leading to immunosuppression in addition to severe economic losses due to impaired growth, death, and excessive condemnations of carcasses because of skeletal muscle hemorrhages [9]. The virus is evolutionarily related to rotaviruses (*Reoviridae*) and picornaviruses (*Picornaviridae*) (Dalton and Rodriguez, [8]). The virus can be adapted to grow and produce cytopathic effects in chicken embryo fibroblasts (CEF) [25].

Nitric oxide (NO) has been shown to inhibit a number of viruses, including Herpes Simplex virus type 1 [7], Ectromelia virus, Vaccinia virus [15], Vesicular Stomatitis virus [5], and murine Friend leukemia retrovirus [1]. Lin et al. [20] have reported the inhibitory effect of NO on Japanese encephalitis viral RNA synthesis, viral protein accumulation, and virus release from infected cells. NO also inhibited the replication cycle of Encephalomyocarditis virus [14], Coxsackie virus [26], Marek’s diseases virus [27], Respiratory Syncytial virus [3] and Severe Acute Respiratory Syndrome virus [2]. NO combines with superoxide radical to produce peroxynitrite radical (ONOO^−^) that reacts with capsid proteins on Coxsackie virus, leading to the inhibition of viral entry into cells [22]. NO also inhibits a variety of transcription factors [13] and viral proteinases [6] that are required for viral replication. Takhampunya et al. [23] reported the inhibitory effect of NO on Dengue virus infection, partly via the inhibition of the RdRp (RNA-dependent RNA polymerase enzyme) activity, which then down-regulates viral RNA synthesis. Jena [17] could demonstrate the inhibition of IBD virus (IBDV) replication in CEF by NO.

Previously, we have shown profound inhibition of IBDV in CEF by root extract of the Indian ginseng, *Withania somnifera* (Linn.) Dunal (WS); however, the mechanism of inhibition was not clear [11]. WS is a well-known inducer of NO. Iuvone et al. [16] found that WS significantly increased NO production in vitro through concentration-dependent up-regulation of inducible nitric oxide synthetase (*i*NOS) expression. Hence, the present investigation was undertaken to ascertain the production of NO as an underlying mechanism of the inhibitory effect of WS against IBDV in CEF.

## Materials and methods

### Cell culture

Nine – Eleven days old, embryonated chicken eggs, were obtained from the Instructional Poultry Farm, G. B. Pant University of Agriculture and Technology, and used for obtaining primary monolayer cultures of CEF as described previously by Villegas [24]. The fibroblasts were resuspended in EMEM with 10% fetal bovine serum, and the cell concentration was adjusted to a final seeding rate of 1 × 10^7^ cells/mL of the media. About 3 mL and 100 μL of the seed were added per well in 6-well and 96-well tissue culture plates, respectively. The plates were incubated at 37 °C with 5% CO_2_ tension for 24 h to obtain the monolayer.

### Virus

Pre-confirmed, CEF-adapted IBD virus of strain UA-Bz 1, passage 6, available in the department was used in this study. Unless stated otherwise, the CEF were infected with the virus at a multiplicity of infection of 0.1.

### *Withania somnifera* Root extract

Methanol: chloroform: water:: 12:5:3 (MCW) extract of roots of *Withania somnifera* roots obtained and characterized as described previously [12] was used in the present study at a concentration of 160 μg/mL pre-determined to be non-cytotoxic to CEF (data not shown).

### Nitric oxide assay

#### Griess test

CEF monolayers were grown in 6-well cell culture plates and upon reaching 80–90% confluence, they were infected with IBDV with or without MCW extract treatment. 0.75 mL of the culture medium was withdrawn from each group at 0, 2, 4, 6, 24 and 48 h and transferred to five wells (150 μL each) of a 96-well plate. The volume withdrawn from the 6-well plate was made up with medium of matching MCW extract concentration. To each well of the 96-well plate, 100 μL of Griess reagent was added and allowed 30 min for color development, following which the plate was read at 570 nm. Concentrations of nitric oxide were expressed in terms of sodium nitrite-equivalents, obtained by using a sodium nitrite standard curve.

#### Effect of aminoguanidine supplementation

Aminoguanidine is a known inhibitor of inducible nitric oxide [21]. First, the non-cytotoxic dose of aminoguanidine for CEF cells was determined by MTT [3-(4,5-dimethythiazol-2-yl)-2,5-diphenyl tetrazolium bromide] assay, as described elsewhere [10]. Thereafter, aminoguanidine, at its non-cytotoxic dose, was used to treat IBDV-infected CEF at −2, 0, 2, 4 or 6 h post-infection (HPI) in the presence or absence of MCW extract. The plate was allowed to incubate and MTT assay was performed 48 HPI.

### Statistical analyses

All statistical operations were performed in MS-Excel 2007. Statistical comparisons between different groups were made by analysis of variance (ANOVA) followed by post hoc Tukey test performed with Daniel’s XL Toolbox *v.*6.53. Unless stated otherwise, all statistical inferences were drawn at *p* < 0.01.

## Results and discussion

**Fig. 1:**
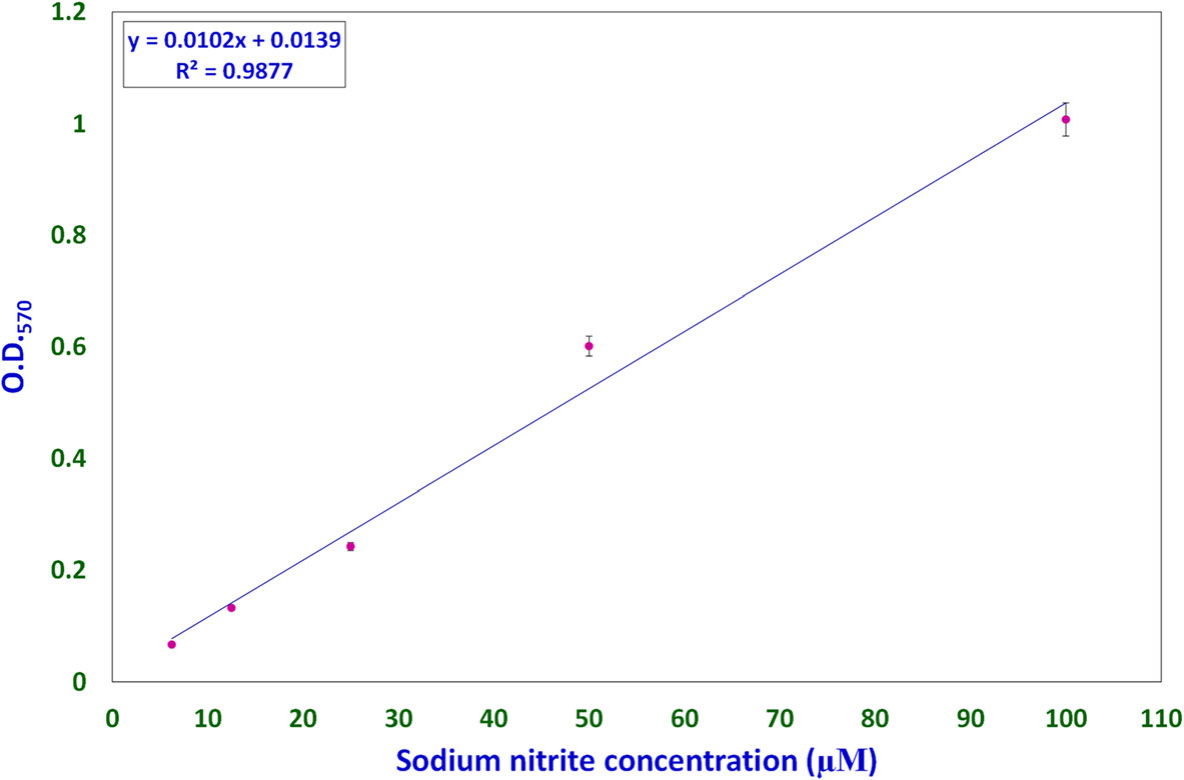
Sodium nitrite standard curve for determination of nitric oxide concentration. Standard curve for determining and expressing nitric oxide concentrations was derived using solutions of known concentration of sodium nitrite. Each concentration of sodium nitrite was assayed thrice independently, and the mean of the three observations was used to obtain the standard curve

**Fig. 2:**
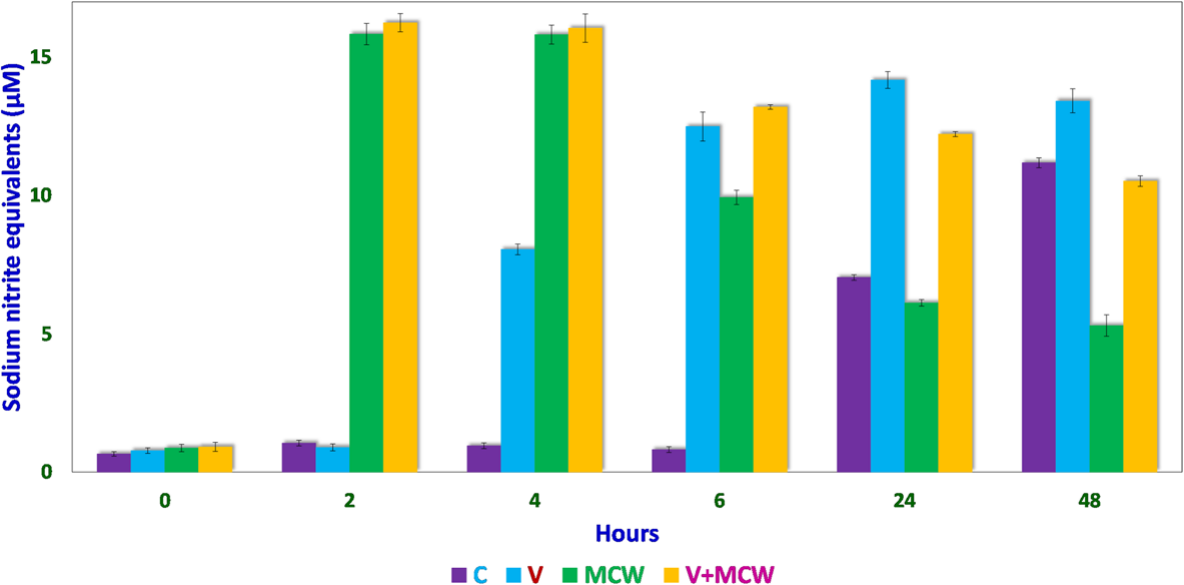
Results of Griess test. Nitric oxide concentrations, expressed as micromolar (μM) sodium nitrite-equivalents, at various intervals of time in control (C), virus-infected (V), uninfected and extract-treated CEF (MCW), and virus-infected and extract-treated (V + MCW) CEF have been shown for comparison

Nitric oxide levels were determined on the basis of a sodium nitrite standard curve (y = 0.0102x + 0.0139, R[2] = 0.9877; Fig. 1) using Griess reagent. Nitric oxide levels, expressed as micromolar equivalents of sodium nitrite, at different time intervals are shown in Fig. 2. MCW extract, alone or with the virus, caused a rapid and significant (*p* < 0.01) increase in NO levels within 2 h of extract supplementation. The increased levels of NO declined significantly faster in the uninfected CEF than in the virus-infected CEF. CEF infected with the virus and left untreated showed a more gradual but persisting increase in NO levels. These findings suggest that MCW extract causes a rapid rise in NO levels in CEF that is inhibitory to the virus. Our findings are supported by previous findings of Jena [17], where it has been shown that NO is inhibitory to IBDV in vitro. It has been stated that IBDV infection alone is insufficient to induce NO production in CEF in vitro; cytokines or chemical donors are essentially required to achieve NO production in CEF [17]. Our results establish that IBDV infection induces production of NO by CEF. Moreover, WS can induce NO in CEF irrespective of IBDV infection. Control CEF also showed a late rise in NO levels; this may possibly have been due to senescence-associated changes.

**Fig. 3:**
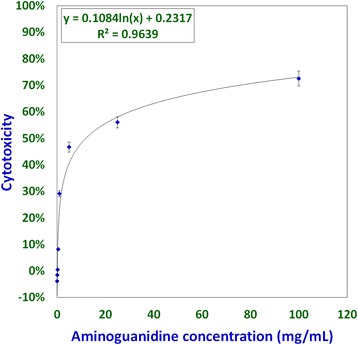
Determination of non-cytotoxic concentration of aminoguanidine for CEF; CEF growing in 96-well plates were treated with different concentrations of aminoguanidine, four wells per concentration. The test was repeated thrice independently and the mean of the values was used for determining the concentration-dependent cytotoxicity of aminoguanidine. The non-cytotoxic dose and median lethal dose were calculated from the standard curve

**Fig. 4:**
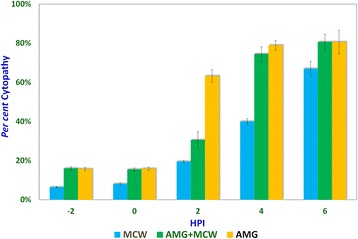
Effect of aminoguanidine supplementation on cytopathy induced by IBDV in CEF; virus-infected CEF were treated with either extract (MCW), aminoguanidine (AMG), or both aminoguanidine and MCW extract (AMG + MCW) at various intervals of time. Percent virus-induced cytopathy at 48 HPI has been shown for comparison

Studies involving aminoguanidine (AMG), a known inhibitor of NO induction, were undertaken to further elucidate the interplay of NO, MCW extract, and IBDV. The non-cytotoxic dose of AMG was determined at 118 μg/mL whereas the *LD*_*50*_ was determined at about 11.88 mg/mL (Fig. 3). For the ease of dilution and dispensing, AMG was supplemented at a concentration of 100 μg/mL that was well within the range of concentrations known to inhibit inducible NO [21]. IBDV-induced cytopathy increased significantly with the delay in MCW supplementation (Fig. 4). The presence of AMG significantly attenuated the virus-inhibitory activity of MCW. These findings of AMG supplementation studies suggest that anti-IBDV activity of MCW extract involves NO production, which gets diminished in the presence of AMG. Nonetheless, MCW extract must be capable of inhibiting IBDV in vitro through additional mechanisms, not necessarily involving induction of NO, as some inhibition of the virus by MCW extract occurred even in the presence of AMG.

It could be seen that presence of AMG early during infection itself was inhibitory to the virus, suggesting that either AMG is capable of exerting direct effects on the virion or that NO is needed early during infection for the pathogenesis of IBDV in vitro. Indeed, NO is required early during infection by IBDV. NO plays a dual role in the pathogenesis of IBD. Infection by IBDV causes the host to produce NO, which initially helps the virus but later turns detrimental to it. Khatri et al. [19] reported that bursal macrophages were susceptible to IBDV infection and macrophage infection was associated with induction of *i*NOS. Macrophages from the infected chicken also showed up-regulated cytokine gene expression and increased production of NO. Such activated macrophages inhibit the proliferation of splenocytes in response to mitogenic stimulation. Inhibition of the mitogenic response is likely mediated by NO and this T-cell suppressive activity helps in virus survival. NO attracts and enhances infiltration of inflammatory cells in the bursa, promoting local tissue damage, which initially helps in the spreading of the virus and later helps in clearing the pathogen [18]. In vivo, the infection with virulent IBDV can result in detectable NO levels in serum and the immunosuppressed chicken that fail to induce NO have more severe disease and a higher degree of virus replication [4].

In conclusion, we show that *Withania somnifera* root extract induces early production of nitric oxide in chicken embryo fibroblasts, which reduces IBDV-induced cytopathy. However, induction of nitric oxide is not the sole mechanism of inhibition of IBDV-induced cytopathy by *Withania somnifera*. Although nitric oxide production by *Withania somnifera* inhibits cytopathy, the inhibition of nitric oxide production, by aminoguanidine, early during infection also inhibits IBDV-induced cytopathy showing the paradoxical essentiality of nitric oxide in the pathogenesis of IBDV.
